# Treating Acute Leukemia During the COVID-19 Pandemic in an Environment With Limited Resources: A Multicenter Experience in Four Latin American Countries

**DOI:** 10.1200/GO.20.00620

**Published:** 2021-04-23

**Authors:** Roberta Demichelis-Gómez, Martha Alvarado-Ibarra, Jule Vasquez-Chávez, Nancy Delgado-López, Cynthia Gómez-Cortés, Karla Espinosa-Bautista, Ana Cooke-Tapia, Andrea Milán-Salvatierra, Andrés Gómez-De León, Yu Ling Lee-Tsai, Daniel Rosales-López, Álvaro Cabrera-García, Fabián Amador-Medina, Alejandra Córdoba-Ramírez, Iván Murrieta-Álvarez, Juan Carlos Solís-Poblano, Elia Apodaca-Chávez, Juan Rangel-Patiño, José Luis Álvarez-Vera, Luara Arana-Luna, José Antonio De la Peña-Celaya, María Eugenia Espitia-Ríos, Eleazar Hernández-Ruiz, Juan Manuel Pérez-Zúñiga, Estefanía Peña-López, Rosa González-Rivera, María Fernanda García-Leyva, Mónica Tejeda-Romero, Jorge Cruz-Rico, Carolina Balderas-Delgado, Guillermo J. Ruíz-Argüelles, David Gómez-Almaguer

**Affiliations:** ^1^ Instituto Nacional de Ciencias Médicas y Nutrición Salvador Zubirán, Mexico City, Mexico; ^2^ Centro Médico Nacional 20 de noviembre, ISSSTE, Mexico City, Mexico; ^3^ Instituto Nacional de Enfermedades Neoplásicas, Lima, Peru; ^4^ Hospital de Especialidades, Centro Médico Nacional siglo XXI, Mexico City, Mexico; ^5^ Instituto Nacional de Cancerología, Mexico City, Mexico; ^6^ Complejo Hospitalario Doctor Arnulfo Arias Madrid, Panama, Panama; ^7^ Hospital Juárez de México, Mexico City, Mexico; ^8^ Universidad Autónoma de Nuevo Léon, Hospital Universitario Dr. José Eleuterio González, Monterrey, Mexico; ^9^ Hospital General San Juan de Dios de Guatemala, Guatemala; ^10^ Instituto Guatemalteco de Seguridad Social, Guatemala City, Guatemala; ^11^ Hospital Regional de Alta Especialidad de Ixtapaluca, Estado de México, Mexico; ^12^ Hospital Regional de Alta Especialidad del Bajío, León, Mexico; ^13^ Centro de Hematología y Medicina Interna Puebla, Puebla, Mexico; ^14^ Hospital de Especialidades CMN “Manuel Ávila Camacho,” IMSS Puebla, Mexico

## Abstract

**PURPOSE:**

The COVID-19 pandemic is a colossal challenge for global health; nonetheless, specific subgroups face considerably higher risks for infection and mortality. Among patients with malignant diseases, those with hematologic neoplasms are at a higher risk for poor outcomes. The objective of this study was to register treatment modifications associated with the COVID-19 pandemic and their short-term consequences in Latin America.

**METHODS:**

Multicenter, prospective, observational, cohort study including patients older than 14 years from 14 centers in four countries (Mexico, Peru, Guatemala, and Panama) who had a confirmed diagnosis of acute leukemia, and who were undergoing active treatment since the first COVID-19 case in each country until the cutoff on July 15, 2020.

**RESULTS:**

We recruited 635 patients. Treatment modifications because of the COVID-19 pandemic were reported in 40.8% of cases. The main reason for such modifications was logistic issues (55.0%) and the most frequent modification was chemotherapy delay (42.0%). A total of 13.1% patients developed COVID-19 disease, with a mortality of 37.7%. Several factors were identified as independently associated with mortality, including a diagnosis of acute myeloid leukemia (odds ratio 2.38 [95% CI, 1.47 to 3.84]; *P* < .001), while the use of telemedicine was identified as a protective factor (odds ratio 0.36 [95% CI, 0.18 to 0.82]; *P* = .014).

**CONCLUSION:**

These results highlight the collateral damage of COVID-19 in oncology patients.

## INTRODUCTION

In December 2019, the WHO was alerted regarding an outbreak of cases of atypical pneumonia documented in China. The etiologic agent, severe acute respiratory syndrome coronavirus 2 (SARS-CoV-2), spread worldwide in the months following and has now derived in a global public health threat. Up to January 3, 2021, more than 84 million cases and two million deaths have been documented worldwide.^1^ Hospital centers around the world were restructured to face this emerging pandemic, prioritizing care for patients with COVID-19, and in turn negatively affecting patients with other diseases who also require care delivered in the hospital setting. In Latin America, as well as other developing nations, the pandemic emerged in an already challenged environment with considerable inequities in terms of healthcare access and availability of resources, as well as a deep economic and social disparity in the population adding additional vulnerabilities when facing the COVID-19 pandemic. Cancer healthcare services in Latin America have significant differences compared with Italy or China where the first COVID-19 outbreaks occurred. Although the incidence of cancer is lower in Latin America, mortality is higher. This is both related to presentation in advanced stages, poorer access to specialized care, and fragmented healthcare systems causing delays.^2^

**CONTEXT**

**Key Objective**
What has been the impact of the modifications in the standard treatment of patients with acute leukemias derived from the COVID-19 pandemic in Latin America?
**Knowledge Generated**
In a cohort of 635 patients, treatment modifications because of the COVID-19 pandemic were reported in 40.8% of cases and the main reason for such modifications was logistic issues. A total of 13.1% patients developed COVID-19 disease, with a mortality of 37.7%, but the main mortality cause during the study period was leukemia.
**Relevance**
These results highlight the collateral damage of COVID-19 in oncology patients and the need to adapt treatment recommendations to the real-world scenario.

Older age and immunocompromise are factors associated with a higher mortality in patients with COVID-19.^3^ Therefore, patients with acute leukemias are a particularly susceptible subgroup. Early reports identified that among patients with cancer, those with hematologic neoplasms have the worse prognosis, with mortality ranging from 28.6% to 37%.^4,5^ Several associations and field experts and key opinion leaders have issued recommendations pertaining to the care and ideal management of patients with leukemias in the context of the COVID-19 pandemic.^6–10^ Nonetheless, in many instances, there are considerable logistic questions as well as availability challenges that are determinant and can affect decision making in the clinical setting.

The objective of this multicenter cohort study was to describe the modifications in the standard care of patients with acute leukemia as well as their short-term clinical outcomes in four Latin American countries. Additionally, we sought to evaluate the frequency and severity of COVID-19 in this patient cohort.

## METHODS

This was a multicenter, prospective cohort study, where collected data from patients in 14 healthcare facilities in four Latin American countries (Mexico, Peru, Panama and Guatemala) were analyzed. The study was approved by the Ethics Committee of the Institutional Review Board and performed in accordance with the Helsinki Declaration. Clinical outcomes were monitored until July 15, 2020, when data cutoff was set for a preplanned analysis. Data were collected from medical records at each facility and deposited in a centralized deidentified database for final analysis. The information was collected by the physicians in charge of treating acute leukemias at each center.

### Patients

We included patients > 14 years of age with the diagnosis of acute leukemia including acute myeloid leukemia (AML), acute lymphoblastic leukemia (ALL), and acute promyelocytic leukemia (APL) confirmed by flow cytometry or immunohistochemistry who were undergoing any type of active treatment at the time the first COVID-19 case was documented in each country (Mexico: February 28, 2020; Peru: March 6, 2020; Panama: March 8, 2020; Guatemala: March 13, 2020). The first case in each country was determined according to the Johns Hopkins coronavirus resource center.

### Outcomes and Definitions

The primary objective was to describe treatment and consult modifications in the context of the COVID-19 pandemic. Secondary outcomes included the development of COVID-19 disease and its complications, as well as cause of death during the study period.

AML was classified as low, intermediate, or high risk according to the European Leukemia Net.^11^ APL was classified as low, intermediate, or high risk as per the Sanz criteria.^12^ ALL was classified as high-risk according to the presence of at least one of the following criteria: hyperleukocytosis (white blood cells > 30 × 10^9^/L) for B-cell ALL, t(v;11q23), complex karyotype, or persistent positive minimal residual disease.^13^ COVID-19 diagnosis was defined as per the WHO case definitions. Confirmed COVID-19 cases were those with a positive laboratory confirmation irrespective of clinical signs and symptoms. Probable COVID-19 disease was considered as a suspect case for which testing for the SARS-CoV-2 virus was either not performed or inconclusive. Suspect-cases were defined as those with acute respiratory illness and history of travel 14 days prior, or contact with a confirmed or probable COVID-19 case, or severe acute respiratory illness in the absence of an alternative diagnosis.^14^ The probable cases were included as COVID-19 cases for analysis. COVID-19 was stratified according to disease severity. COVID-19 severe disease was characterized by a respiratory rate > 30 breaths per minute, O_2_ saturation < 93%, PaO_2_:FiO_2_ < 300 mm Hg, and infiltrate progression > 50% in 24-48 hours. Critical disease was defined as that which presented with organ failure, septic shock, or respiratory failure. COVID-19 disease was classified as mild-moderate in the absence of the aforementioned criteria for severe or critical disease.

COVID-19–related deaths were considered if SARS-CoV-2 infection was the direct cause of death, regardless of the leukemia status. Leukemia-related deaths were from leukemia progression, without COVID-19.

### Statistical Considerations

Continuous variables were described as medians and interquartile ranges, and categorical variables were summarized as proportions. Medians were compared using the Mann-Whitney *U* test. The χ^2^ test and Fisher's exact test were used for assessment of statistical significance of categorical variables, determined as a value for *P* < .05. To identify risk factors, we calculated the odds ratio (OR) and performed a logistic regression, which included those variables that were statistically significant or clinically relevant (*P* < .05). All analyses were performed using the SPSS software (version 22).

## RESULTS

### Baseline Characteristics

A total of 635 patients met the inclusion criteria and were included in the study; among these, 66.6% were from Mexico, 20% from Peru, 7.2% from Guatemala, and 6.1% from Panama. Most patients (91.8%) were treated in centers also designated to treat patients with COVID-19, whereas 40.2% and 15.4% were treated in centers where elective hospitalization and emergency services, respectively, were suspended because of the reconversion of health services derived from the COVID-19 pandemic.

Among the included patients, 58.1% had a diagnosis of Philadelphia-negative ALL, 25.7% had a diagnosis of AML, 9% had a diagnosis of APL, and 7.2% had Philadelphia-positive ALL. A total of 50.4% of patients included were male, and median age was 35 years (range: 14-90 years). Although patients presented across all ages, only 10.1% of the study population were > 60 years of age. Patients diagnosed with AML were significantly older (median 41 years) compared with those diagnosed with ALL (median 31 years) (*P* > .01). In terms of risk categories, most patients were considered high-risk patients (58.8%) and 68.3% were in complete remission, undergoing consolidative or maintenance therapy, whereas 14.5% of the sample represented newly diagnosed patients and 17.2% were patients with relapsed or refractory disease (Table 1).

**TABLE 1:**
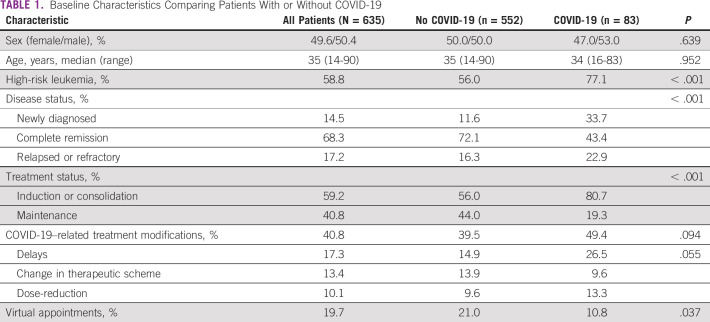
Baseline Characteristics Comparing Patients With or Without COVID-19

### Treatment Modifications in the Context of the COVID-19 Pandemic

The COVID-19 pandemic led to treatment modifications in 40.8% of the cases. Treatment modifications were performed in 38.4% and 42.1% of patients with AML or APL and ALL, respectively (*P* = .396), and were significantly more frequent in patients undergoing induction or consolidation compared with maintenance therapy (45.5% *v* 34.0%; *P* = .004). Reasons for modifications in treatment protocols included logistic limitations (55.0%), medical decision (37.0%), and patient choice (8.1%). However, the reason for treatment modification was significantly different when comparing patients with AML and ALL diagnoses, in which logistic limitations were cited for treatment modifications in 44.0% of patients with AML compared with 59.9% of patients with ALL. Similarly, medical decision (52.3% *v* 29.7%) and patient choice (3.6% *v* 10.2%) were also differently distributed among patients with AML and ALL diagnoses, respectively (*P* = .002).

**FIG 1:**
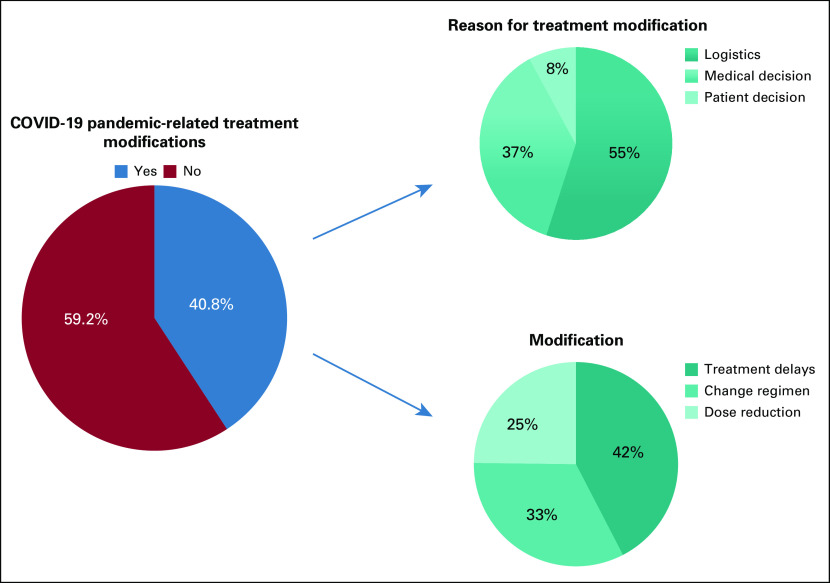
COVID-19 pandemic-related treatment modifications.

In terms of the modifications in treatment regimens, chemotherapy delay was the most frequently recorded (42.0%), followed by regimen modification (33.0%) and dose-reductions (25.0%). This information is summarized in Figure 1.

Forty-five patients with ALL were newly diagnosed (median age of 33 years); treatment modifications occurred in 26.7% and the main modification was delaying the treatment in 58.4%. By contrast, 47 patients with AML were newly diagnosed (median age of 42 years); treatment modifications occurred without significant differences between younger and older than 60 years (46.2% *v* 25%; *P* = .437) and the main modification was dose-reductions in 50% of the cases.

Furthermore, an allogeneic stem-cell transplant (AlloHSCT) was planned in 25.2% of the included subjects and postponed in 72.5% of them. When considering ALL (N = 115), 82.6% where considered high-risk patients and AlloHSCT was postponed in 70% versus 80% in low- or intermediate-risk (*P* = .584). By contrast, AlloHSCT was planned in 45 patients and 71.1% were high-risk patients. Again, there was no significant difference in the percentage of cases in which AlloHSCT was postponed according to risk: 62.5% in high-risk versus 69.2% in low- or intermediate-risk (*P* = .743).

Virtual appointments were implemented in 19.7% of cases, which included consults by telephone (77.7%), by email (19.8%), or by videoconference (2.5%). During this follow-up period, 4.1% of the patients abandoned treatment.

### COVID-19 Disease

During follow-up, 83 patients (13.1%) developed COVID-19. The majority had mild-moderate disease (54.2%), whereas 45.8% were classified as having either severe or critical illness. Invasive mechanical ventilation was required in 27.7% of the cases. When considering baseline characteristics, we identified that patients with active leukemia (newly diagnosed or relapsed) were significantly more likely to develop COVID-19 disease (OR 3.46 [95% CI, 2.16 to 5.5]; *P* < .001). Other factors associated with a higher risk for developing COVID-19 included having high-risk leukemia (OR 1.63 [95% CI, 1.54 to 4.52]; *P* < .001) and being treated in a center where elective hospitalization was possible (OR 2.17 [95% CI, 1.29 to 3.67]; *P* = .004). Conversely, the implementation of virtual appointments was significantly associated with a reduced risk for developing COVID-19 (OR 0.46 [95% CI, 0.22 to 0.94]; *P* = .037). Treatment modifications and longer intervals between appointments were not associated with a reduced risk for developing COVID-19.

### Clinical Outcomes and Mortality

Among patients with acute leukemia and COVID-19 disease, mortality rate was 37.7%, representing 4.9% of the entire study population. In Table 2, we show the characteristics of the patients with COVID-19, comparing those who died from the disease compared with those who did not.

**TABLE 2:**
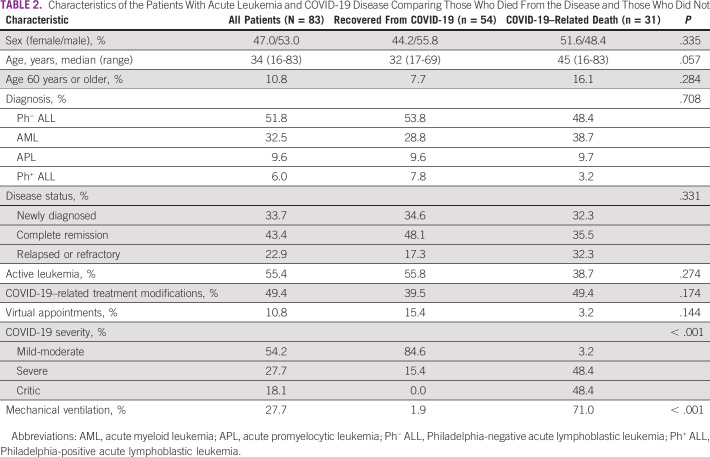
Characteristics of the Patients With Acute Leukemia and COVID-19 Disease Comparing Those Who Died From the Disease and Those Who Did Not

We did not identify any significant differences in terms of COVID-19 mortality for the patients in this study as per leukemia diagnosis (AML 42.9% *v* ALL 33.3%; *P* = .491), age (older than 60 years 55.5% *v* < 60 years 35.1%; *P* = .284), disease activity (active leukemia 41.3% *v* 32.4% nonactive disease; *P* = .495), or in patients receiving induction or consolidation (38.8%) compared with those undergoing maintenance therapy (31.3%; *P* = .775).

Among patients in complete remission, 11.3% relapsed. There was no significant association regarding treatment modifications, longer intervals between appointments, or virtual consults with the risk for a relapse. Patients who developed COVID-19 had a numerically higher rate for disease relapse compared with those who did not develop COVID-19; however, this difference did not reach statistical significance (18.8% *v* 10.3%; OR 2.01 [95% CI, 1.00 to 4.00]; *P* = .057).

A total of 16.7% of the patients included in the cohort had died by the data lockdown date (July 15, 2020). The main cause of death was leukemia progression (57.7%), followed by COVID-19 disease (29.2%) and treatment-related mortality (13.2%). Several factors were independently associated with mortality, including AML versus ALL diagnosis (OR 2.38 [95% CI, 1.47 to 3.84]; *P* < .001), relapsed-refractory disease (OR 7.62 [95% CI, 4.61 to 12.60]; *P* > .001), and induction or consolidation versus maintenance therapy (OR 2.54 [95% CI, 1.47 to 4.37]; *P* = .001), while being followed with virtual consults (OR 0.36 [95% CI, 0.18 to 0.83]; *P* = .014) was protective (Table 3).

**TABLE 3:**
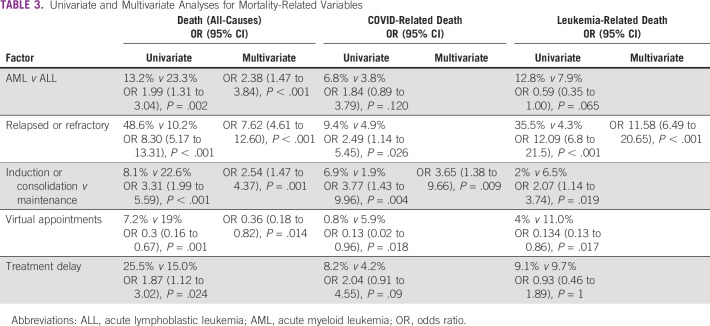
Univariate and Multivariate Analyses for Mortality-Related Variables

## DISCUSSION

There is a considerable amount of information regarding the clinical outcomes of patients with hematologic malignancies who develop COVID-19. Meanwhile, the impact of the pandemic in terms of treatment modifications in patients with acute leukemia has not been previously established.

The focus of existing recommendations is on reducing the risk for nosocomial transmission of SARS-CoV-2 by reducing hospital visits and the prioritization of ambulatory treatment whenever possible. Other key points include a more liberal approach to the use of growth factors, routine use of PCR testing for SARS-CoV-2 before intensive chemotherapy, treatment modifications for those cases in which prognosis will not be negatively affected and last, postponement of consolidative AlloHSCT in patients who are not considered at a high risk for relapse.^6–10^ The actual field-expert recommendations favor the use of standard induction chemotherapy in newly diagnosed fit patients.^9,10^

In our study, we show that treatment modifications occurred in almost half of the population. Also, despite the youth in our population, modifications to induction chemotherapy were performed very frequently. Unlike what is recommended, the transplants that were planned were mostly in high-risk patients and the majority were postponed. Importantly, we report that hospital capacity limitations and not the medical criteria are the most frequent cause for treatment modification.

Interestingly, the results also show that in this patient cohort, a dose-reduction or treatment postponement was not associated with a reduced risk for developing COVID-19.

Our cohort has particular characteristics such as a very young median age and a predominance of ALL. This is consistent with the epidemiology previously reported in our acute leukemia population and is very characteristic of Central America and other developing countries.^15^ Despite the young age, the frequency of COVID-19 was considerably higher (13.1%) compared with data described in other populations. Other prospective cohorts have reported a COVID-19 incidence of < 1%, in other regional settings.^16,17^ It is important to consider that we include only public healthcare centers; therefore, it is a cohort of patients with a low socioeconomic level. It has been described in other populations that the educational status and the socioeconomic status are associated with a higher risk and worse outcomes with COVID-19 and these may contribute to the high rate of COVID-19 and the high mortality.^18,19^

Mortality for COVID-19 patients was 37.7%, a high mortality rate that is in accordance with previous studies in Italy and China, where they reported mortality ranging from 50% to 65%.^20,21^ Furthermore, an Italian retrospective cohort identified a higher standardized mortality ratio (2.04 [95% CI, 1.77 to 2.34]) when comparing patients with hematologic cancers who developed COVID-19 compared with the general Italian population with COVID-19.^22^

An interesting finding in our study is the fact that although only 19.7% of the patients were followed through virtual resources, a significant benefit was observed both in terms of risk of developing COVID-19 as well as in mortality. The use of telemedicine has proven feasible in other types of malignancies. A previous study collected information from a sarcoma clinic in the United Kingdom, where 316 patients received medical consults through telemedicine resources. Importantly, patients who mainly required face-to-face appointments include those requiring intravenous chemotherapy, with progressive disease, or who required a performance status (Eastern Cooperative Oncology Group) evaluation. The authors report a high level of satisfaction compared with face-to-face and 80% of the patients desired having telemedicine options for their future care.^23^ Another study from Turkey, where the majority of the patients were receiving hormone therapy, oral targeted therapy, or chemotherapy, showed that problems were solved without requiring a hospital visit in 93% of cases.^24^ Contrary to other malignancies, patients with acute leukemia can reap lesser benefit from this virtual healthcare modality, since the majority of patients require either intravenous therapy and/or transfusions in the hospital setting. There is also the possibility that the least ill patients and the younger ones with more access to technologic resources are the ones who most frequently received virtual follow-up and this may be reflected in better outcomes. Nonetheless, the convincing evidence for the utility of telemedicine in the current setting is promising and combined with the advent of targeted agents could be feasible in specific patient subgroups.^25^

Some of the limitations to be able to generalize the results of this study are the short follow-up and the differences in recruitment across countries.

In conclusion, the collateral damage of the COVID-19 pandemic has been very important among patients with leukemia in Latin American countries and surely in the rest of the world. We have to adapt the management recommendations in this population and use telemedicine tools to limit the damage.

## Data Availability

A data sharing statement provided by the authors is available with this article at DOI https://doi.org/10.1200/GO.20.00620.
